# Genomic analysis of eight clinical *Rothia* isolates

**DOI:** 10.1128/mra.00153-25

**Published:** 2025-08-13

**Authors:** Shannon R. West, Aanuoluwa E. Adekoya, Sydney K. Arriaga, Swetha Rajagopol, Samantha L. Varriale, Paul J. Planet, Carolyn B. Ibberson

**Affiliations:** 1 Department of Microbiology, University of Tennessee 189504 , Knoxville, Tennessee, USA; 2 School of Biological Sciences, University of Oklahoma 6187, Norman, Oklahoma, USA; 3 Department of Neuroscience, The University of Arizona 8041, Tucson, Arizona, USA; 4 Department of Pediatrics, University of Pennsylvania and Children's Hospital of Philadelphia 6572, Philadelphia, Pennsylvania, USA; University of Maryland School of Medicine, Baltimore, Maryland, USA

**Keywords:** genome analysis, *Rothia*, human microbiome

## Abstract

Here, we report the genome sequences of eight clinical isolates of *Rothia*, seven of which were isolated from the upper respiratory tract of people with cystic fibrosis (pwCF). Analyzing the genomes of members of the respiratory microbiome in pwCF can elucidate possible interactions among microbial community members.

## ANNOUNCEMENT

*Rothia* spp. are prevalent and transcriptionally active members of the microbial community found in cystic fibrosis (CF) sputum (1–4). However, the role of *Rothia* in the context of CF respiratory disease remains unknown. Therefore, we analyzed the genomes of eight clinical *Rothia* isolates, seeking to characterize *Rothia* associated with CF.

Oral swabs (oropharynx) were collected at Columbia University Medical Center under IRB approval (#IRBAAAE8112) from three participants in the Ecology of Cystic Fibrosis (Eco-CF) project (5) and preserved at −80°C in either glycerol or dimethyl sulfoxide. The preserved oral swabs were struck onto Heart Infusion agar plates containing lincomycin (5 µg/mL) and colistin sulfate (10 µg/mL) incubated at 37°C, both anaerobically and aerobically, for 3 days. Colonies with characteristic *Rothia* morphology were selected and subcultured to purity. Seven of the eight isolates were obtained by the aforementioned procedure, while one isolate (ILR0003) was obtained through BEI Resources, NIAID, NIH as part of the Human Microbiome Project (6). For DNA extraction purposes, each strain was grown overnight (~17 h) at 37°C with shaking in 10 mL BBL Brain Heart Infusion (BHI; Becton, Dickinson and Co) broth. Cells were harvested by centrifugation and washed 3× with Dulbecco’s PBS (Thermo Fisher Scientific). Genomic DNA was extracted using the Monarch Spin gDNA Extraction kit following the manufacturer’s protocol for Gram-positive bacteria (New England Biolabs). DNA was sent to SeqCoast Genomics (Portsmouth, NH, US), where DNA library preparation and sequencing were performed. Briefly, the DNA was sheared and size selected through the on-bead tagmentation process (7) used for library prep by SeqCoast genomics with the Illumina DNA Prep Tagmentation kit and unique dual indexes. Sequencing was performed on the Illumina NextSeq2000 platform using a 300-cycle flow cell kit, producing 2 × 150 bp paired reads. PhiX control (1%–2%) was spiked into the run to support optimal base calling. Read demultiplexing, read trimming, and run analytics were performed using DRAGEN v3.10.12 (8), a NextSeq2000 on-board analysis software. The Kbase platform was used for genome analyses (9). After quality checking the reads with FastQC v0.12.1 (10), genomes were assembled using SPAdes v3.15.3 (11) and checked for completeness using CheckM v1.0.18 (12). Genomes were annotated using the NCBI Prokaryotic Genome Annotation Pipeline (13). Assembled genomes were quality assessed using QUAST v4.4 (14). Each isolate was identified through KBase using the whole genome sequences of all isolates and SpeciesTree v2.2.0 (15). Default parameters were used except where otherwise noted.

Table 1 contains genome information for each isolate including total reads generated, N50, and coverage. Of the eight isolates, three were identified as *R. mucilaginosa* and five were identified as *R. dentocariosa*. The genomes ranged in length from 2,284,086 to 2,579,644 bp with GC content between 53.65 and 59.53%. The number of NCBI PGAP-predicted genes ranged from 1,806 to 2,239, with *R. dentocariosa* strains containing more genes than *R. mucilaginosa* strains.

**TABLE 1:** *Rothia* isolate metadata, accession links, and genome assembly statistics

Source	HMP	ECOCF0017	ECOCF0017	ECOCF0017	ECOCF0017	ECOCF0024	ECOCF0041	ECOCF0041
Isolate	ILR0003	ILR0004	ILR0005	ILR0006	ILR0007	ILR0008	ILR0010	ILR0011
Taxonomy	*R. mucilaginosa*	*R. mucilaginosa*	*R. dentocariosa*	*R. dentocariosa*	*R. dentocariosa*	*R. dentocariosa*	*R. mucilaginosa*	*R. dentocariosa*
SRA accession	SRR31931841	SRR31931840	SRR31931839	SRR31931838	SRR31931837	SRR31931836	SRR31931835	SRR31931834
WGS accession	JBKQAZ000000000	JBKQBA000000000	JBKQBB000000000	JBKQBC000000000	JBKQBD000000000	JBKQBE000000000	JBKQBF000000000	JBKQBG000000000
Total reads	1,816,161	2,700,816	2,588,374	3,056,208	2,700,534	2,777,206	3,033,056	2,668,962
Length (bp)	2,293,984	2,307,667	2,523,791	2,523,853	2,524,264	2,579,644	2,284,086	2,481,324
Contigs	30	10	10	8	11	22	12	9
GC (%)	59.43	59.53	53.66	53.66	53.66	53.65	59.48	53.77
Genes (#)	1,812	1,815	2,195	2,194	2,196	2,239	1,806	2,139
N50	162,261	630,408	1,446,065	1,446,010	497,918	343,652	708,691	391,089
Coverage	119x	176x	154x	182x	160x	161x	199x	161x
Completeness (%)	99.36	98.12	99.34	99.34	99.34	99.34	98.12	99.34

## Data Availability

The raw sequencing paired-end reads and assembled whole-genome sequence (WGS) for each isolate were deposited in the Sequence Read Archive (SRA) and GenBank databases, respectively. The SRA and WGS accession numbers and links are listed in Table 1. Raw sequences, genome assemblies, and PGAP annotation files can be found under BioProject number PRJNA1208058.
